# Cadmium Toxicity Is Regulated by Peroxisome Proliferator-Activated Receptor δ in Human Proximal Tubular Cells

**DOI:** 10.3390/ijms23158652

**Published:** 2022-08-03

**Authors:** Chikage Mori, Jin-Yong Lee, Maki Tokumoto, Masahiko Satoh

**Affiliations:** Laboratory of Pharmaceutical Health Sciences, School of Pharmacy, Aichi Gakuin University, 1-100 Kusumoto-cho, Chikusa-ku, Nagoya 464-8650, Japan; ag193a01@dpc.agu.ac.jp (C.M.); leejy@dpc.agu.ac.jp (J.-Y.L.); maki@dpc.agu.ac.jp (M.T.)

**Keywords:** cadmium, kidney, *PPARD*, apoptosis

## Abstract

Cadmium (Cd) is a toxic heavy metal that is widely present in the environment. Renal proximal tubule disorder is the main symptom of Cd chronic poisoning. Our previous study demonstrated that Cd inhibits the total activities of peroxisome proliferator-activated receptor (PPAR) transcription factors in human and rat proximal tubular cells. In this study, we investigated the involvement of PPAR in Cd renal toxicity using the HK-2 human proximal tubular cell line. Among PPAR isoform genes, only *PPARD* knockdown significantly showed resistance to Cd toxicity in HK-2 cells. The transcriptional activity of PPARδ was decreased not only by *PPARD* knockdown but also by Cd treatment. DNA microarray analysis showed that *PPARD* knockdown changed the expression of apoptosis-related genes in HK-2 cells. *PPARD* knockdown decreased apoptosis signals and caspase-3 activity induced by Cd treatment. *PPARD* knockdown did not affect the intracellular Cd level after Cd treatment. These results suggest that PPARδ plays a critical role in the modification of susceptibility to Cd renal toxicity and that the apoptosis pathway may be involved in PPARδ-related Cd toxicity.

## 1. Introduction

Cadmium (Cd) is an environmentally toxic metal that adversely affects various tissues such as kidney, liver, and lung [1,2]. Because Cd has a long biological half-life (15–30 years), it accumulates in the kidney and liver upon chronic exposure through dietary intake of contaminated rice, vegetables, fish, and shellfish [1,2]. Several decades ago, many areas in Japan were affected by Cd contamination caused by mining, and long-term Cd ingestion causes renal toxicity [3]. Moreover, it is well known that *itai-itai* disease was caused by Cd concentration in Toyama, Japan [3]. Proximal tubular cells are the primary target of Cd-induced renal toxicity. Cd causes cell death through necrosis, apoptosis, autophagy, disruption of cell–cell adhesions, and production of reactive oxygen species in various tissues, including mouse kidney, and cultured cells [4,5,6]. Among the toxic pathways, cell death of proximal tubular cells of the kidney via the apoptotic pathway is one of the major events of Cd-induced nephrotoxicity.

Our recent studies show that changes in transcriptional activity may be involved in Cd renal toxicity [7,8,9]. Cd decreases the activity of transcription factor MEF2A in HK-2 human proximal tubular cells [9]. MEF2A regulates the expression of GLUT4. Cd decreases the cellular levels of GLUT4 and the transportation of glucose into cells. The decreased glucose level affects ATP production, which causes cytotoxicity in Cd-treated HK-2 cells [9].

Our previous studies demonstrated that Cd inhibits the activities of peroxisome proliferator-activated receptor (PPAR) transcription factors in human and rat proximal tubular cells [8,10]. PPARs belong to the nuclear receptor superfamily of transcription factors and have three isoforms (PPARα, PPARδ, and PPARγ) in humans. PPARs form heterodimers with the retinoid X receptor (RXR) and bind to specific DNA elements [11,12,13]. PPAR affects lipid metabolism, regulation of glucose homeostasis, cell proliferation, differentiation, and apoptosis. PPARα is abundant in tissues with active fatty acid conversion, such as the liver, heart, and digestive tract, and has functions such as fatty acid metabolism and anti-inflammatory actions [14]. PPARγ is mainly expressed in adipose tissue and the immune system, which functions in adipocyte differentiation and insulin regulation [14]. PPARδ is present in various tissues, such as the small intestine, colon, liver, and even cancer tissues; moreover, it regulates metabolism, inflammation, and cell proliferation [14,15,16,17].

As described above, PPARs play diverse and important physiological roles. Furthermore, we have demonstrated that Cd changed the transcriptional activity of PPAR. However, the roles of PPAR in the Cd renal toxicity has not been elucidated. Therefore, this study investigated the effect of PPAR knockdown on Cd toxicity and the role of PPAR in Cd-induced apoptosis of HK-2 cells.

## 2. Results

### 2.1. Identification of PPAR Affecting Cd Toxicity in HK-2 Cells

**Figure 1:**
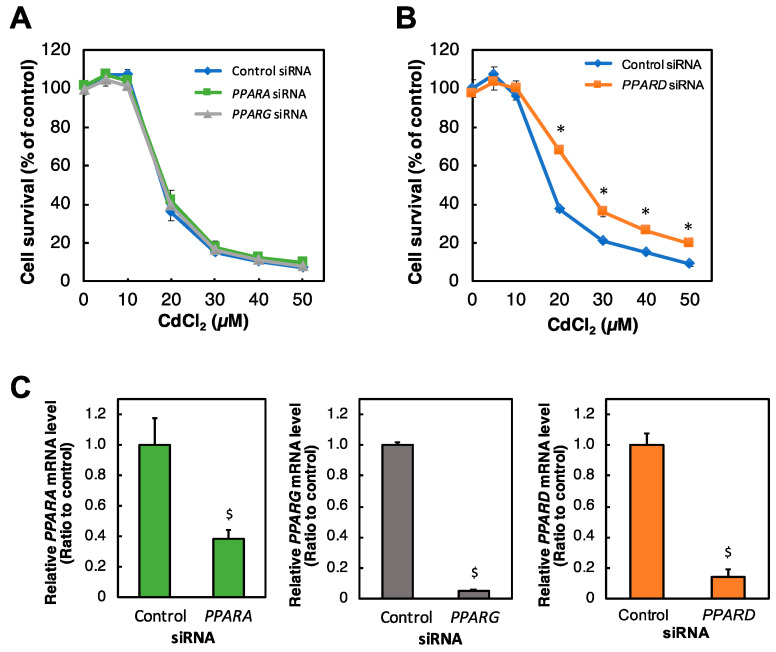
Effect of *PPAR* knockdown on the survival rate of HK-2 cells treated with Cd. (**A**,**B**) After treatment with *PPARA*, *PPARG* or *PPARD* siRNA for 48 h, HK-2 cells were treated with Cd for 24 h. Cell survival was examined by an Alamar blue assay. Values are means ± SD (*n* = 3). * Significantly different from the control siRNA group, *p* < 0.05. (**A**) *PPARA* siRNA and *PPARG* siRNA. (**B**) *PPARD* siRNA. (**C**) Efficiency of *PPARA, PPARG,* and *PPARD* knockdown was examined after HK-2 cells were treated with siRNA against the gene of *PPARA*, *PPARG* or *PPARD* for 48 h. *PPARA*, *PPARG*, and *PPARD* mRNA levels were examined by real-time RT-PCR and normalized to *GAPDH* mRNA levels. Values are means ± SD (*n* = 3). ^$^ Significantly different from the control group, *p* < 0.05.

To investigate the involvement of PPAR in Cd toxicity in HK-2 cells, we examined the effect of knockdown of each *PPAR* gene on Cd toxicity. In human cells, PPARs consist of PPARα (*PPARA*), PPARδ (*PPARD*), and PPARγ (*PPARG*). Among the isoforms, only *PPARD* knockdown significantly conferred resistance to Cd toxicity (Figure 1A,B). Additionally, each siRNA against *PPAR* genes significantly reduced their mRNA levels in HK-2 cells (Figure 1C).

### 2.2. Effects of Cd and PPARD Knockdown on the PPARδ Transcriptional Activity in HK-2 Cells

**Figure 2:**
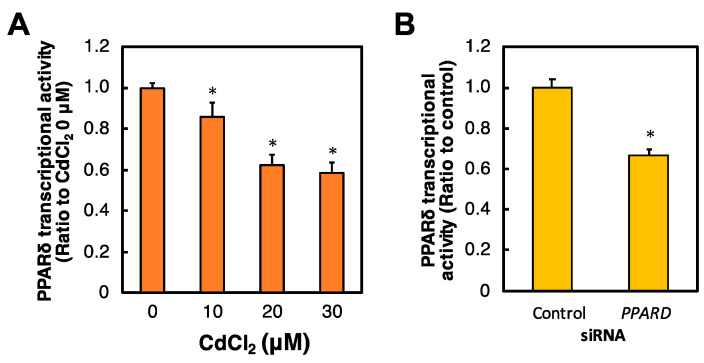
Effect of Cd and *PPARD* knockdown on the PPARδ transcriptional activity in HK-2 cells. (**A**) HK-2 cells were treated with Cd for 6 h. PPARδ transcriptional activity was examined by a PPAR delta Transcriptional Factor Assay Kit after Cd treatment for 6 h. Values are means ± SD (*n* = 3). * Significantly different from the control group, *p* < 0.05. (**B**) PPARδ transcriptional activity by *PPARD* knockdown was examined after HK-2 cells were treated with *PPARD* siRNA for 48 h. Values are means ± SD (*n* = 3). * Significantly different from the control siRNA group, *p* < 0.05.

To investigate the transcriptional activity of PPARδ by exposure to Cd and *PPARD* knockdown, we examined PPARδ transcriptional activity in Cd-treated or *PPARD* knockdown HK-2 cells. Cd treatment for 6 h significantly and dose-dependently decreased PPARδ transcriptional activity in HK-2 cells (Figure 2A). PPARδ transcriptional activity was significantly decreased after *PPARD* knockdown in HK-2 cells (Figure 2B).

### 2.3. Effect of Cd on the PPARD mRNA Level in HK-2 Cells

**Figure 3:**
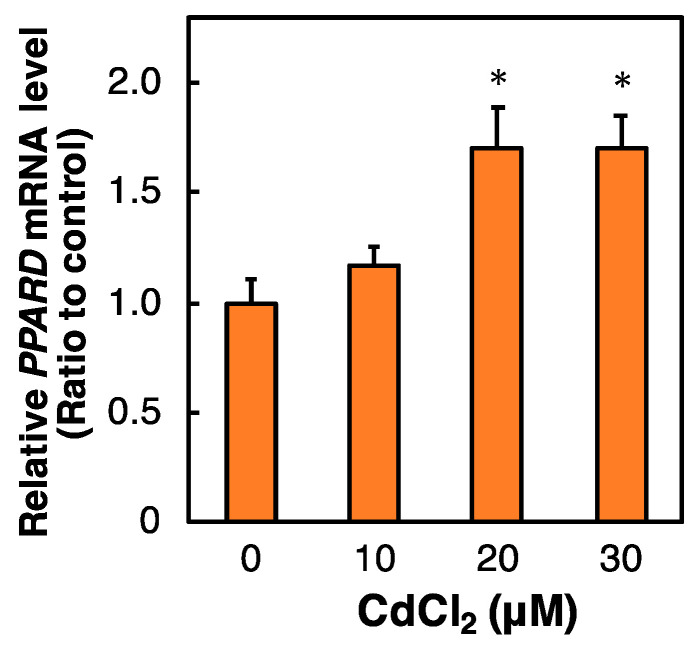
Effect of Cd on the *PPARD* mRNA level in HK-2 cells. HK-2 cells were treated with Cd for 6 h. *PPARD* mRNA levels were examined by real-time RT-PCR after Cd treatment for 6 h. *PPARD* mRNA levels were normalized to *GAPDH* mRNA levels. Values are means ± SD (*n* = 3). * Significantly different from the control group, *p* < 0.05.

To clarify the effect of Cd treatment on *PPARD* expression in HK-2 cells, the *PPARD* mRNA level was examined after HK-2 cells were treated with Cd. Cd treatment for 6 h significantly and dose-dependently increased the *PPARD* mRNA level in HK-2 cells (Figure 3). This result implies that the gene expression of *PPARD* may not contribute to the Cd-inhibited transcriptional activity of PPARδ in HK-2 cells.

### 2.4. Effect of RXR Knockdown on the Viability of HK-2 Cells Treated with Cd

**Figure 4:**
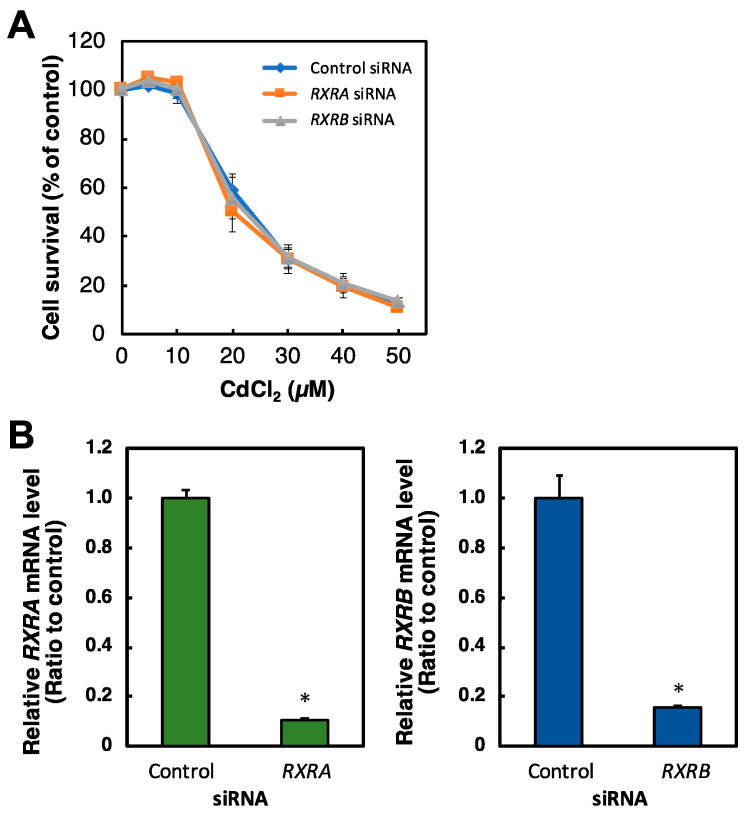
Effect of *RXR* knockdown on the survival rate of HK-2 cells treated with Cd. (**A**) After treatment with siRNA against the gene of *RXRA* or *RXRB* for 48 h, HK-2 cells were treated with Cd for 24 h. Cell survival was examined by the Alamar blue assay. Values are means ± SD (*n* = 3). * Significantly different from the control siRNA group, *p* < 0.05. (**B**) Efficiency of *RXRA* and *RXRB* knockdown was examined after HK-2 cells were treated with siRNA the gene of *RXRA* or *RXRB* for 48 h. *RXRA* and *RXRB* mRNA levels were examined by real-time RT–PCR and normalized to *GAPDH* mRNA levels. Values are means ± SD (*n* = 3). * Significantly different from the control group, *p* < 0.05.

PPAR forms a dimer with RXR, and therefore resistance to Cd toxicity by *PPARD* knockdown may be associated with RXR activity. To investigate the involvement of RXR in the resistance Cd toxicity by *PPARD* knockdown in HK-2 cells, we examined the effect of *RXR* knockdown on Cd toxicity. RXR also consists of three isoforms [11,12,13]. Cd cytotoxicity in *RXRA* and *RXRB* knockdown cells was similar to that in control cells (Figure 4A). We also confirmed that *RXRA* and *RXRB* siRNA treatments significantly reduced the levels of *RXRA* and *RXRB* mRNAs in HK-2 cells (Figure 4B). Therefore, RXR may be independent of *PPARD* knockdown-decreased sensitivity to Cd toxicity in HK-2 cells.

### 2.5. Identification of Genes Regulated by PPARD Knockdown in HK-2 Cells

**Figure 5:**
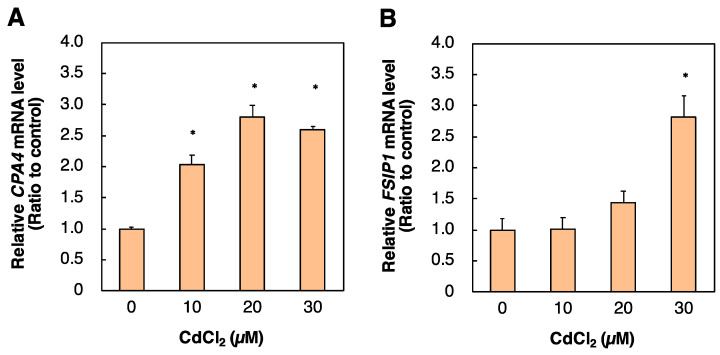
Effect of Cd on *CPA4* and *FSIP1* mRNA levels in HK-2 cells. HK-2 cells were treated with Cd for 6 h. *CPA4* and *FSIP1* mRNA levels were examined by real-time RT–PCR after Cd treatment for 6 h (**A**,**B**). *CPA4* and *FSIP1* mRNA levels were normalized to *GAPDH* mRNA levels. Values are means ± SD (*n* = 3). * Significantly different from the control group, *p* < 0.05.

PPARδ regulates gene expression by transcriptional activity, and therefore PPARδ-related Cd toxicity may affect downstream factor(s) of PPARδ. To identify genes regulated by PPARδ, we performed DNA microarray analysis of HK-2 cells transfected with PPARD siRNA. PPARD knockdown increased the expression of 53 genes by more than three-fold (Table 1). Among the genes, *RYR2*, *ITPK1*, *PALD1*, *ZNF488*, *TFF2*, *IL9R*, *PANX2*, *CPA4*, *CCL19*, *FSIP1*, and *MLXIPL* are involved in apoptosis [18,19,20,21,22,23,24,25,26,27,28]. *PPARD* knockdown decreased the expression of 39 genes by less than or equal to 0.5-fold (Table 2). Among these genes, *LPAR3*, *GAL3ST1*, *PTPN11*, and *RORA* are involved in apoptosis [29,30,31,32]. We examined the effect of Cd on the expression of 15 of the above genes (Figure 5, data not shown). Cd increased the mRNA levels of *CPA4* and *FSIP1* whose expression was induced by *PPARD* knockdown (Figure 5). Increases in cellular levels of CPA4 and FSIP1 negatively act on apoptosis signals [20,23]. These results indicated that apoptosis was associated with resistance to Cd toxicity by *PPARD* knockdown.

### 2.6. Involvement of PPARD Knockdown in Cd-Induced Apoptosis

**Figure 6:**
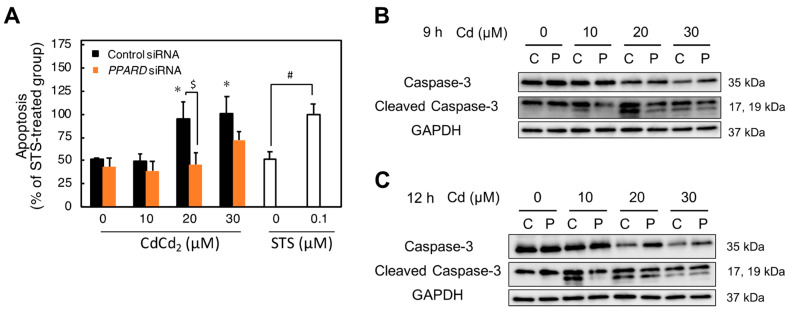
Effect of *PPARD* knockdown on apoptosis of HK-2 cells treated with Cd. (**A**) After treatment with *PPARD* siRNA for 48 h, HK-2 cells were treated with Cd for 12 h. HK-2 cells were treated with STS, as an apoptosis inducer, for 12 h. Apoptosis was examined by a Cell Death Detection ELIZA^PLUS^ Kit after Cd and STS treatment for 12 h. Values are means ± SD (*n* = 3). * Significantly different from the control siRNA + Cd 0 μM group, *p* < 0.05. ^$^ Significantly different from the control siRNA group, *p* < 0.05. ^#^ Significantly different from the STS 0 μM group, *p* < 0.05. (**B**,**C**) After treatment with *PPARD* siRNA for 48 h, HK-2 cells were treated with Cd for 9 h (**B**) or 12 h (**C**). Protein levels of caspase-3 and cleaved caspase-3 were examined by western blotting after Cd treatment for 9 or 12 h. GAPDH was used as the loading control.

Previous studies have demonstrated that Cd induces apoptosis in HK-2 cells [4,7,8,33]. Furthermore, some downstream factors of PPARδ are involved in the apoptosis pathway [34,35]. Therefore, we examined whether the resistance to Cd toxicity by *PPARD* knockdown was involved in apoptosis. To compare apoptosis levels, staurosporine (STS) was used as an apoptosis inducer. The apoptosis was significantly induced in control cells by 20 and 30 µM Cd treatment for 12 h. Additionally, Cd-induced apoptosis was significantly inhibited by *PPARD* knockdown. The apoptosis induced by 20 and 30 µM Cd treatment was similar to that induced by 0.1 µM STS treatment for 12 h (Figure 6A). Induction of apoptosis by Cd is mediated through caspase-3 activation in Cd renal toxicity [4]. Therefore, we investigated whether inhibition of Cd-induced apoptosis by *PPARD* knockdown was involved in caspase-3 activation. Cd treatment (10–30 µM) for 9 h increased the level of cleaved caspase-3, whereas *PPARD* knockdown decreased cleaved caspase-3 increased by Cd (Figure 6B). The treatment with 10 µM Cd for 12 h markedly increased the level of cleaved caspase-3 and *PPARD* knockdown decreased the increased one by Cd treatment (Figure 6C). However, the increased level of cleaved caspase-3 by 20 and 30 µM Cd for 12 h was not affected by *PPARD* knockdown. These results indicated that *PPARD* knockdown partly protected HK-2 cells from the Cd-induced apoptosis through a decrease in the cleaved caspase-3 level.

### 2.7. Effect of PPARD Knockdown on the Intracellular Cd Concentration

**Figure 7:**
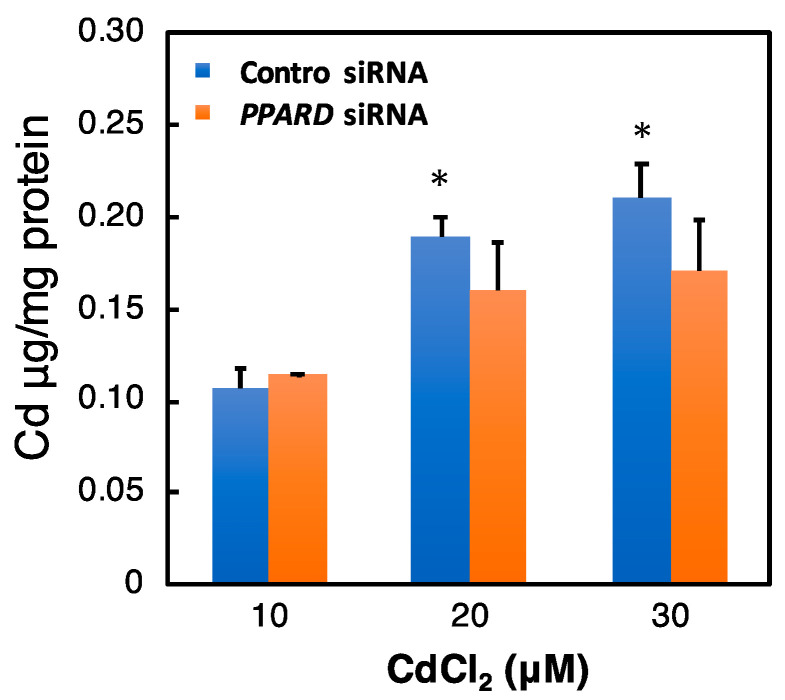
Effect of *PPARD* knockdown on the intracellular Cd concentration. After treatment with *PPARD* siRNA for 48 h, HK-2 cells were treated with Cd for 12 h. The Cd concentration was measured by an atomic absorption spectrometer. Values are means ± SD (*n* = 3). * Significantly different from the control siRNA + Cd 10 μM group, *p* < 0.05.

*PPARD* knockdown may affect the Cd accumulation in HK-2 cells. Therefore, we investigated the effect of *PPARD* knockdown on the intracellular Cd concentration after Cd treatment. The intracellular Cd level was increased dose-dependently on the treatment concentration (Figure 7). However, *PPARD* knockdown did not affect the intracellular Cd level (Figure 7).

## 3. Discussion

This study strongly suggests that the transcription factor PPARδ modifies susceptibility to Cd toxicity. Additionally, PPARδ transcriptional activity is inhibited in response to Cd stimulation in HK-2 cells. Furthermore, the decrease in PPARδ activity inhibits the apoptosis pathway induced by Cd in HK-2 cells and *PPARD* knockdown is resistant to Cd toxicity in HK-2 cells. The inhibitory effect of Cd on the transcriptional activity of PPARδ may be a biological reaction to protect against Cd toxicity. Moreover, *PPARD* knockdown did not change the concentration of Cd in cells, suggesting that *PPARD* knockdown is not involved in Cd uptake and excretion in HK-2 cells.

Cd increased the level of *PPARD* mRNA. However, transcriptional activity of PPARδ was suppressed by Cd. Therefore, the induction of gene expression of *PPARD* is unlikely to be associated with the suppression of PPARδ transcriptional activity. Several research groups have reported on the regulation of PPARδ activity. In the skeletal muscle, it has been reported that the activity of PPARδ is regulated by AMPK, CRYs, and PGC [36,37]. AMPK promotes PPARδ-dependent transcription. However, AMPK does not increase the PPARδ phosphorylation. AMPK may be present in a transcriptional complex with PPARδ [37]. CRY1 and CRY2 can selectively repress the transcriptional activity of PPARδ [36]. In addition, NCOA6 deficiency suppresses the activity of PPARδ in human and mouse hearts [38,39]. Recent studies demonstrated that Cd changes the AMPK-related pathway and disrupts the expressions of CRY1 [40,41]. These findings suggest that Cd may affect the transcriptional activity of PPARδ via the various mechanisms including the interaction with the cofactors and PPARδ.

Individual differences have been observed in the onset of chronic renal toxicity caused by Cd. Previous studies reported that there are gene polymorphisms in *PPARD* [42,43]. Our study demonstrated that PPARδ is a modification factor in Cd renal toxicity because *PPARD* knockdown is resistant to Cd renal toxicity. The presence of gene polymorphisms in *PPARD* means that there is a population with low PPARδ levels, which may be a population less sensitive to Cd renal toxicity. Moreover, changes in PPARδ may be one of the factors that cause individual differences in the development of Cd renal toxicity. Therefore, these findings suggest PPARδ plays an important role as a modification factor against Cd renal toxicity.

Cd decreases the activities of transcription factors YY1 and FOXF1. As a result, gene expression of the UBE2D family—downstream factors of YY1 and FOXF1—decreases in HK-2 cells [7]. Suppression of UBE2D family expression causes accumulation of apoptosis-inducing factor p53 in the cells, which induces apoptosis [7,33]. Cd decreases transcriptional activity of ARNT in HK-2 cells [8]. The cellular level BIRC3, a downstream factor of ARNT, was decreased by Cd treatment. BIRC3 is an inhibitor of apoptosis. Cd-decreased BIRC3 also induces apoptosis in the HK-2 cells [8]. PPARγ is involved in Cd-induced apoptosis and oxidative stress in renal epithelial cells and hepatocytes [44,45]. In the rat heart cells, DHA (docosahexaenoic acid), which acts as a ligand for PPARδ, has been reported to promote apoptotic cell death, increase caspase-3 activity, and reduce Akt phosphorylation via PPARδ [46]. Furthermore, it has been reported that intracellular prostacyclin promotes apoptosis by activating PPARδ in the human kidney cells [47]. On the other hand, it has also been reported that in mouse brain, PPARδ activation suppresses caspase-3 activation through miR-15a and its downstream Bcl-2 and protects cerebrovascular vessels by reducing apoptosis [48]. We demonstrated that PPARδ is a crucial factor that influences Cd-induced apoptosis in human proximal tubular cells. These findings indicate that Cd triggers renal toxicity via an apoptosis pathway with associations between various related factors. Depending on the various physiological circumstances, there may be differences in the contribution of each apoptosis-related factor to Cd-induced renal toxicity.

## 4. Materials and Methods

### 4.1. Cell Culture and Treatment

HK-2 cells purchased from the American Type Culture Collection (Manassas, MA, USA) were cultured in Dulbecco’s modified Eagle’s medium/Ham’s F-12 nutrient mixture (Sigma-Aldrich, St. Louis, MO, USA) containing 10% fetal bovine serum (Gibco, Grand Island, NY, USA), 25 U/mL penicillin (DS Pharm, Osaka, Japan), 25 μg/mL streptomycin (DS Pharm), 1% insulin-transferrin-selenium X (Gibco), 10 ng/mL epidermal growth factor (Sigma-Aldrich), and 5 ng/mL hydrocortisone at 37 °C in a humidified incubator with 5% CO_2_. The cells were cultured in test plates at a density of 250 cells/mm^2^ for 48 h. After discarding the culture medium, the cells were treated with Cd (CdCl_2_; 98.0%; Fujifilm Wako Pure Chemical Co., Tokyo, Japan) in serum-free culture medium.

### 4.2. Small Interfering RNA (siRNA) Transfection

Silencer Select Predesigned siRNAs against human *PPAR* and *RXR* mRNAs were purchased from Ambion (Grand Island, NY, USA). Control siRNA (Silencer Select Negative Control #1 siRNA) was also purchased from Ambion. siRNA transfection was performed with Lipofectamine RNAiMAX (Invitrogen, Grand Island, NY, USA). After the siRNA mixture was incubated for 15 min with Lipofectamine RNAiMAX and Opti-MEM I Reduced Serum Medium (Opti-MEM; Gibco), HK-2 cells were transfected with the siRNA mixture (1 nM siRNA per sequence, 0.2% Lipofectamine RNAiMAX, and 10% Opti-MEM) for 48 h.

### 4.3. Cell Survival Rate

HK-2 cells were treated with the siRNA mixture in 96-well plates for 48 h. After treatment, Alamar blue (10%; Invitrogen) was added and the cells were incubated for 4 h at 37 °C. Fluorescence was measured at an excitation wavelength of 540 nm and emission wavelength of 595 nm with a SpectraMax^®^ iD3 microplate reader (Molecular Devices, San Jose, CA, USA).

### 4.4. RNA Extraction

HK-2 cells were treated with the siRNA mixture and Cd in 6-well plates. Cd-treated HK-2 cells were washed twice with ice-cold phosphate-buffered saline (PBS(−); Nissui, Tokyo, Japan). Total RNA was extracted with a PureLink^TM^ RNA Mini Kit (Ambion) in accordance with the manufacturer’s instructions. RNA quantity and purity were measured using a BioSpec-nano spectrophotometer (Shimadzu, Kyoto, Japan).

### 4.5. DNA Microarray Analysis

DNA microarray analysis was performed by Hokkaido System Science Co., Ltd. (Sapporo, Japan). Complementary RNA (cRNA) was synthesized from 50 ng total RNA using a Low Input Quick Amp Labeling Kit (Agilent Technologies, Santa Clara, CA, USA). HK-2 cells were treated with the siRNA mixture in 6-well plates for 48 h. The total RNAs were pooled from the independent three samples. Double-stranded cDNA from control siRNA- or *PPARD* siRNA-treated cells was transcribed in the presence of cyanine (Cy) 3-CTP or Cy5-CTP, respectively. These two sets of labeled cRNAs (300 ng each) were mixed and hybridized to a SurePrint G3 Human 8 × 60 K ver. 3.0 (Agilent Technologies) by a Gene Expression Hybridization Kit (Agilent Technologies) for 17 h at 65 °C. Fluorescent images were recorded with the Agilent Microarray Scanner (G2600D). Digitized image data were processed with Agilent Feature Extraction ver. 12.0.3.1. Information on each gene was obtained from the National Center for Biotechnology Information database.

### 4.6. Real-Time Reverse Transcription (RT)-PCR

To generate cDNA, total RNA was subjected to a PrimeScript RT Reagent Kit (Perfect Real Time) (Takara Bio, Shiga, Japan). Real-time PCR was performed with SYBR^®^ Premix Ex Taq^TM^ II (Perfect Real Time) (Takara Bio) on the Thermal Cycler Dice Real Time System (Takara Bio). The thermal cycling conditions were 10 s at 95 °C followed by 40 cycles of 5 s at 95 °C and 30 s at 60 °C. Gene expression was normalized to *GAPDH* mRNA levels. The oligonucleotide sequences of the primers used were as follows: sense, 5′-GAAACAGGCCTTCTCAGTGC-3′ and antisense, 5′-TTGCTGGGTCGTCTTTTTCT-3′ for the human *PPARD* gene; sense, 5′-ACGATTCGACTCAAGCTGGT-3′ and antisense, 5′-GTTGTGTGACATCCCGACAG-3′ for the human *PPARA* gene; sense, 5′-TTCAGAAATGCCTTGCAGTG-3′ and antisense, 5′-CCAACAGCTTCTCCTTCTCG-3′ for the human *PPARG* gene; sense, 5′-CAAGGACTGCCTGATTGACA-3′ and antisense, 5′-CTGGTCGACTCCACCTCATT-3′ for the human *RXRA* gene; sense, 5′-CCTGAGGGCAATCATTCTGT-3′, and antisense, 5′-CCTGCTGCTCAGGGTACTTC-3′ for the human *RXRB* gene; sense, 5′-GCACCGTCAAGGCTGAGAAC-3′ and antisense, 5′-TGGTGAAGACGCCAGTGGA-3′ for the human *GAPDH* gene.

### 4.7. Western Blot Analysis

HK-2 cells were treated with the siRNA mixture and Cd in 6 cm dishes. After treatment, the cells were washed twice with ice-cold PBS(−) and harvested in RIPA buffer (25 mM Tris, pH 7.6, 150 mM NaCl, 1% NP-40, 1% sodium deoxycholate, and 0.1% sodium dodecyl sulfate (SDS); Thermo Fisher Scientific, Waltham, MA, USA). Protein concentrations were measured by Pierce^TM^ BCA Protein Assay Kit (Thermo Fisher Scientific). Protein samples were separated on an SDS-polyacrylamide gel and transferred to a polyvinylidene difluoride membrane. The membrane was probed with primary antibodies and then with a horseradish peroxidase-conjugated secondary antibody (1:10,000 dilution; GE Healthcare, Little Chalfont, UK). The proteins were detected by enhanced chemiluminescence using Chemi-Lumi One Super (Nacalai Tesque, Kyoto, Japan). Chemiluminescence images were acquired with the ChemiDoc^TM^ imaging system (BIO-RAD, Hercules, CA, USA). The primary antibodies were anti-GAPDH (1:1000 dilution) from American Research Products (Waltham, MA, USA), and anti-Caspase-3 (1:1000 dilution) and anti-Cleaved Caspase-3 (diluted 1:1000) from Cell Signaling Technology (Danvers, MA, USA).

### 4.8. Nuclear Protein Extraction

Nuclei were extracted with a Nuclear Extraction Kit (ab113474; abcam, Tokyo, Japan). HK-2 cells were treated with Cd or the siRNA mixture in 10 cm dishes. The treated cells were pooled from the independent two samples. After treatment, HK-2 cells were washed twice with ice-cold PBS(−) and harvested in PBS(−). The cells were shaken at 200× *g* for 10 min at 4 °C in Pre-extraction Buffer that included a Protease Inhibitor Cocktail and dithiothreitol (DTT). The cytoplasmic fraction was collected by centrifugation at 14,000× *g* for 3 min at 4 °C. The nuclear pellet was resuspended in Extraction Buffer, which included the Protease Inhibitor Cocktail and DTT, and incubated at 4 °C for 1 h with agitation every 15 min. The mixture was centrifuged at 16,000× *g* for 10 min at 4 °C, and the supernatant was collected. Protein concentrations were measured by Pierce^TM^ BCA Protein Assay Kit.

### 4.9. PPARδ Transcriptional Activity Assay

PPARδ transcriptional activity was determined by a PPAR delta Transcriptional Factor Assay Kit (ab133106; abcam). After treatment, nuclei were extracted from HK-2 cells. Complete Transcription Factor Binding Assay Buffer (CTFB) was prepared by adding Transcription Factor Binding Assay Buffer, Reagent A, and DTT. Nuclei and CTFB were added to 96-well plates. After covering the 96-well plates with the included cover, the plates were incubated overnight at 4 °C without agitation. The 96-well plates were washed five times with Wash Buffer. Transcription Factor PPAR delta Primary Antibody was prepared by adding Transcription Factor Antibody Binding Buffer (ABB) and PPAR delta Primary Antibody. After completely removing the Wash Buffer, Transcription Factor PPAR delta Primary Antibody was added to the 96-well plates. After covering the 96-well plates with the included cover, the plates were incubated for 1 h at room temperature without agitation. The 96-well plates were then washed five times with Wash Buffer. Transcription Factor Goat Anti-Rabbit HRP conjugate was prepared by adding ABB and Goat Anti-Rabbit HRP conjugate. After completely removing the Wash Buffer, Transcription Factor Goat Anti-Rabbit HRP conjugate was added to the 96-well plates. After covering the 96-well plates with the included cover, the plates were incubated for 1 h at room temperature without agitation. The 96-well plates were washed five times with Wash Buffer. After completely removing the Wash Buffer, Transcription Factor Developing Solution was added to the 96-well plates. The 96-well plates were incubated for 45 min at room temperature with gentle agitation while protected from light. Absorbance at 450 nm was read within 5 min after adding Stop Solution to the 96-well plate with an iMark^TM^ Microplate Reader (BIO-RAD).

### 4.10. Apoptosis Assay

Apoptosis was examined by a Cell Death Detection ELIZA^PLUS^ Kit (Roche, Basel, Switzerland). HK-2 cells were treated with the siRNA mixture and Cd in 96-well plates. After treatment, the HK-2 cells were washed twice with ice-cold PBS(−). Lysis Buffer was added to the cells, followed by incubation for 30 min at room temperature to lyse the cells. The cell lysates were repeatedly pipetted and then transferred to 96-well plates. Incubation buffer containing Anti-Histone Biotin and Anti-DNA POD was added to the 96-well plates. After covering with a close contact cover, the mixture was incubated at room temperature for 2 h while shaking at 300 rpm. The 96-well plate was washed three times with Incubation Buffer and then the Incubation Buffer was completely removed. ABTS solution, in which ABTS tablets were dissolved in the Substrate Buffer, was placed in a 96-well plate and incubated for 20 min with shaking at 250 rpm. After adding the ABTS stop solution, the absorbance was measured at 405 nm and 490 nm with the SpectraMax^®^ iD3 microplate reader. Staurosporine (STS) was used for the positive control treatment [49]. The degree of Cd-induced apoptosis was normalized to apoptosis induced by 0.1 µM STS treatment (12 h).

### 4.11. Determination of Cd Content

After siRNA or Cd treatment using 6-well plates, cells were washed twice with ice-cold PBS(−) and then three times with PBS(−) containing 2 nM ethylene glycol tetraacetic acid (Nacalai Tesque). The cells were then harvested in 1 mL RIPA buffer and digested with nitric acid and hydrogen peroxide. After sample digestion, metal analysis was carried out using an atomic absorption spectrometer (200 series AA; Agilent Technologies). Protein concentrations were measured by Pierce^TM^ BCA Protein Assay Kit to normalize the Cd content.

### 4.12. Statistical Analysis

Statistical analyses were performed by one- or two-way ANOVA. When the *F*-value was significant (*p* < 0.05), Bonferroni’s multiple *t*-test was performed for *post-hoc* comparison (*p* < 0.05). Statistical analyses were performed with SPSS Statistics (IBM, Tokyo, Japan).

## Figures and Tables

**Table 1:** Genes with expression increased by *PPARD* knockdown (>3-fold).

Gene	Accession Number	Description	Fold Change
*RYR2*	NM_001035	Ryanodine receptor 2	5.75
*CDHR2*	NM_017675	Cadherin related family member 2	5.31
*PCDH19*	NM_020766	Protocadherin 19	4.92
*TMEM184A*	NM_001097620	Transmembrane protein 184A	4.91
*GLYAT*	NM_005838	Glycine-N-acyltransferase	4.89
*AUTS2*	NM_001127231	Activator of transcription and developmental regulator AUTS2	4.86
*MSR1*	NM_002445	Macrophage scavenger receptor 1	4.80
*CRLF2*	NM_001012288	Cytokine receptor like factor 2	4.77
*SLC23A3*	NM_144712	Solute carrier family 23 member 3	4.76
*IL27*	NM_145659	Interleukin 27	4.71
*PSG2*	NM_031246	Pregnancy specific beta-1-glycoprotein 2	4.70
*PITPNM3*	NM_031220	PITPNM family member 3	4.46
*ITPK1*	NM_014216	Inositol-tetrakisphosphate 1-kinase	4.42
*ACTN2*	NM_001278344	Actinin alpha 2	4.39
*BTBD16*	NM_001318189	BTB domain containing 16	4.38
*PALD1*	NM_014431	Phosphatase domain containing paladin 1	4.38
*RGS11*	NM_003834	Regulator of G protein signaling 11	4.34
*SMCO1*	NM_001077657	Single-pass membrane protein with coiled-coil domains 1	4.29
*ZNF488*	NM_153034	Zinc finger protein 488	4.21
*GAL3ST1*	NM_004861	Pentatricopeptide repeat domain 2	4.20
*CACNG7*	NM_031896	Calcium voltage-gated channel auxiliary subunit gamma 7	4.19
*SERPINF1*	NM_002615	Serpin family F member 1	4.19
*ESPNL*	NM_194312	Espin like	4.19
*LONRF2*	NM_198461	LON peptidase N-terminal domain and ring finger 2	4.19
*PDE4C*	NM_001330172	Phosphodiesterase 4C	4.18
*PTPN11*	NM_002834	Transmembrane protein 225B	4.16
*PRDM11*	NM_001359633	PR/SET domain 11	4.11
*TFF2*	NM_005423	Trefoil factor 2	4.11
*PDZD4*	NM_032512	PDZ domain containing 4	4.02
*CHRNE*	NM_000080	Cholinergic receptor nicotinic epsilon subunit	4.01
*LRRC3*	NM_030891	Leucine rich repeat containing 3	4.00
*IL9R*	NM_176786	Interleukin 9 receptor	3.94
*PANX2*	NM_001160300	Pannexin 2	3.88
*CPA4*	NM_016352	Carboxypeptidase A4	3.76
*PLCB1*	NM_182734	Phospholipase C beta 1	3.65
*TLDC2*	NM_080628	TBC/LysM-associated domain containing 2	3.63
*PPCDC*	NM_134260	Phosphopantothenoylcysteine decarboxylase	3.61
*RGMB*	NM_001366509	Repulsive guidance molecule BMP co-receptor b	3.51
*PDE4DIP*	NM_001350522	Phosphodiesterase 4D interacting protein	3.51
*RNF125*	NM_017831	Ring finger protein 125	3.50
*JRK*	NM_001279352	Jrk helix-turn-helix protein	3.50
*TPRX1*	NM_198479	Tetrapeptide repeat homeobox 1	3.44
*HAAO*	NM_012205	3-Hydroxyanthranilate 3,4-dioxygenase	3.41
*SLC43A1*	NM_003627	Solute carrier family 43 member 1	3.35
*CCL19*	NM_006274	C-C motif chemokine ligand 19	3.29
*RBM20*	NM_001134363	RNA binding motif protein 20	3.23
*FSIP1*	NM_152597	Fibrous sheath interacting protein 1	3.19
*MPZL3*	NM_198275	Myelin protein zero like 3	3.18
*SMKR1*	NM_001195243	Small lysine rich protein 1	3.12
*MLXIPL*	NM_032951	MLX interacting protein like	3.11
*PLXNC1*	NM_005761	Plexin C1	3.11
*OR6T1*	NM_001005187	Olfactory receptor family 6 subfamily T member 1	3.10
*EGF*	NM_001178130	Epidermal growth factor	3.06

**Table 2:** Genes with expression decreased by *PPARD* knockdown (≤0.5-fold).

Gene	Accession Number	Description	Fold Change
*TTLL9*	NM_001035	Tubulin tyrosine ligase like 9	0.10
*TNF*	NM_000594	Tumor necrosis factor	0.23
*SAA1*	NM_000331	Serum amyloid A1	0.24
*CCL20*	NM_004591	C-C motif chemokine ligand 20	0.25
*FLG*	NM_002016	Filaggrin	0.26
*PPARD*	NM_006238	Peroxisome proliferator activated receptor delta	0.32
*LPAR3*	NM_012152	Lysophosphatidic acid receptor 3	0.35
*PCOTH*	NM_001348114	Pro-X-Gly collagen triple helix like repeat containing	0.36
*HACD3*	NM_016395	3-Hydroxyacyl-CoA dehydratase 3	0.38
*ANKS1B*	NM_001352196	Ankyrin repeat and sterile alpha motif domain containing 1B	0.39
*TRAK2*	NM_015049	Trafficking kinesin protein 2	0.40
*ST3GAL6*	NM_006100	ST3 beta-galactoside alpha-2,3-sialyltransferase 6	0.40
*LTB*	NM_014216	Lymphotoxin beta	0.40
*UHMK1*	NM_175866	U2AF homology motif kinase 1	0.40
*DDR1*	NM_001202522	Discoidin domain receptor tyrosine kinase 1	0.41
*KCNS3*	NM_014431	Potassium voltage-gated channel modifier subfamily S member 3	0.42
*SAA2*	NM_030754	Serum amyloid A2	0.42
*FSIP1*	NM_152597	Fibrous sheath interacting protein 1	0.43
*EFHC1*	NM_153034	EF-hand domain containing 1	0.43
*GAL3ST1*	NM_004861	Galactose-3-O-sulfotransferase 1	0.43
*IL18R1*	NM_003855	Interleukin 18 receptor 1	0.44
*SGCB*	NM_000232	Sarcoglycan beta	0.44
*PCDH20*	NM_022843	Protocadherin 20	0.44
*CA9*	NM_001216	Carbonic anhydrase 9	0.44
*ZDHHC11B*	NM_001351303	Zinc finger DHHC-type containing 11B	0.44
*PTPN11*	NM_002834	Protein tyrosine phosphatase non-receptor type 11	0.45
*CXADR*	NM_001338	CXADR Ig-like cell adhesion molecule	0.46
*YES1*	NM_005423	YES proto-oncogene 1, Src family tyrosine kinase	0.46
*LOC391322*	NM_032512	D-dopachrome tautomerase-like	0.47
*CCDC146*	NM_020879	Coiled-coil domain containing 146	0.47
*EIF1AY*	NM_004681	Eukaryotic translation initiation factor 1A Y-linked	0.47
*PTX3*	NM_176786	Pentraxin 3	0.47
*PTGFRN*	NM_001160300	Prostaglandin F2 receptor inhibitor	0.47
*CSF2*	NM_016352	Colony stimulating factor 2	0.48
*ANKRD37*	NM_181726	Ankyrin repeat domain 37	0.48
*ENTPD2*	NM_001246	Ectonucleoside triphosphate diphosphohydrolase 2	0.49
*RORA*	NM_134260	RAR related orphan receptor A	0.49
*PACC1*	NM_018252	Proton activated chloride channel 1	0.50
*CD83*	NM_004233	CD83 molecule	0.50
